# High incidence of fracture events in patients with Long-Gap Esophageal Atresia (LGEA): A retrospective review prompting implementation of standardized protocol^☆^

**DOI:** 10.1016/j.bonr.2015.06.002

**Published:** 2015-06-14

**Authors:** Sigrid Bairdain, Brenda Dodson, David Zurakowski, Lawrence Rhein, Brian D. Snyder, Melissa Putman, Russell W. Jennings

**Affiliations:** a Department of Pediatric Surgery, Boston Children's Hospital, Harvard Medical School, Boston, MA, United States; b Department of Pharmacy, Boston Children's Hospital, Harvard Medical School, Boston, MA, United States; c Division of Critical Care Medicine, Department of Anesthesia, Perioperative and Pain Medicine, Boston Children's Hospital, Harvard Medical School, Boston, MA, United States; d Department of Pulmonology, Boston Children's Hospital, Harvard Medical School, Boston, MA, United States; e Department of Orthopedics, Boston Children's Hospital, Harvard Medical School, Boston, MA, United States; f Division of Endocrinology, Department of Medicine, Boston Children's Hospital, Harvard Medical School, Boston, MA, United States

## Abstract

**Purpose:**

To identify factors associated with an increased risk of fractures in Long-Gap Esophageal Atresia (LGEA) patients. Following implementation of a risk-stratified program, we hypothesized a reduction in fracture incidence within this potentially high-risk population.

**Methods:**

A retrospective review of LGEA-patients admitted between 2005 and 2014 was conducted. Symptomatic fractures with radiographic confirmation were defined as events. Univariate and multivariable analysis evaluated factors including admission weight-for-age z-score, primary versus secondary Foker process (FP), weight at Foker Stage I, days and episodes of paralysis, number of parenteral nutrition (PN) days, cumulative dose of loop diuretics adjusted for body weight and days exposed, and exposure to non-loop diuretics. A fracture-prevention protocol was initiated in 2012; incidence was evaluated pre and post-intervention.

**Results:**

Fifty-nine patients met inclusion criteria. Twenty-three (39%) patients in the entire cohort incurred at least one fracture during their hospitalization utilizing the Foker process. Given this high percentage, a targeted fracture-prevention protocol was initiated in 2012. Fracture incidence decreased from 48% prior to the protocol to 21% following the protocol (*P* = 0.046). Several variables that were associated with an increased risk of fractures on univariate analysis included prior esophageal anastomosis attempt (*P* = 0.008), number of separate episodes of paralysis (*P* = 0.002), exposure to non-loop diuretics (*P* = 0.006), cumulative loop diuretic dose (*P* < 0.001), as well as cumulative loop diuretic over days exposed (*P* < 0.001). Intensive care unit (ICU) stay (*P* = 0.002) and total length of hospitalization (*P* < 0.001) were also significantly longer among patients with a fracture. Number of separate episodes of paralysis was the only independent risk factor for the development of a fracture; patients having more than 3 episodes of paralysis had an estimated risk of fracture 15 times higher than those patients paralyzed only once or twice (O.R. 15.87, 95% C.I.: 1.47–171.23, *P* = 0.008).

**Conclusion:**

Episodes of paralysis appeared to be the most significant risk factor for fractures in patients with LGEA who underwent the Foker procedure. The incidence of symptomatic fractures decreased significantly following implementation of a standardized protocol in this series of LGEA patients with continued prospective evaluation.

## Highlights

• LGEA patients who undergo the Foker process may be at an increased risk for fractures.
• We examined the risk factors for fractures in our surgical population.
• We implemented a risk-stratified fracture prevention program in this population that decreased fractures.

## 1 Introduction

Long-Gap Esophageal Atresia (LGEA) is a rare congenital anomaly and frequently requires utilization of interventions that may expose infant patients to increased fracture risk (David and O'Callaghan, 1975, Bairdain et al., 2014, Bairdain et al., In Press). LGEA is often defined by a distance between the upper and a lower atretic esophageal segment of greater than three vertebral bodies (Gross, 1953, Foker et al., 1997). This distance ultimately delineates the timing and ease of repair. Foker et al. (1997) described a unique repair strategy that utilizes external traction sutures to promote in vivo growth of the esophagus through tension-induced natural lengthening followed by delayed primary repair (Foker et al., 1997). During this Foker process, patients typically required prolonged mechanical ventilation, pharmacological paralysis, sedation and analgesia, and utilization of central venous catheters (CVCs) to facilitate medication and parenteral nutrition (PN) administration (Bairdain et al., 2014). The degree and length of exposure to these aforementioned entities is proportional to the difficulty of the repair. As a referral center for LGEA, Boston Children's Hospital (BCH) provides a unique environment to evaluate the potential effects of exposure to certain interventions and the development of inpatient fractures (Bairdain et al., In Press). Therefore, we aimed to identify factors associated with increased risk of fractures in LGEA patients undergoing the Foker process. Following implementation of a risk-stratified fracture prevention program, we also hypothesized that there would be a reduction in fracture incidence within this high-risk population.

## 2 Methods and materials

### 2.1 Population and definitions

Following the approval of our institutional review board (IRB), we retrospectively reviewed the medical records of all patients managed utilizing the Foker process for LGEA from 2005 to 2014 at our institution. Esophageal atresia (EA) patients with or without tracheoesophageal fistula (TEF) were considered to have LGEA when primary anastomosis was not possible because of length of the gap between the upper and lower esophageal segments (Al-Shanafey and Harvey, 2008). All other forms of EA as well as those who did not undergo the Foker process (FP) were excluded. Primary LGEA patients were those patients who did not undergo a previous operation or whose previous operations were limited to a gastrostomy placement while those patients who had esophageal surgery elsewhere were considered secondary FP cases (Bairdain et al., In Press).

During the FP, patients were intubated, sedated and muscle relaxed during external traction. Patient received parental nutrition (PN) while they were nil per os (NPO). There was not a standardized regimen for when and what types of diuretics were utilized; this treatment was at the discretion of the treatment team. We categorized diuretics into loop and non-loop diuretics groups. We determined use of non-loop diuretics as a dichotomous variable based on any versus no use. Since loop diuretic use was more common and has a greater association with fractures, we normalized the comparative doses of the various loop diuretics. The approximate dose equivalency used for this evaluation was bumetanide 1 milligram (mg) = furosemide 40 mg = torsemide 20 mg = ethacrynic acid 50 mg (Lexicomp).

Symptomatic fractures were defined as incident events when radiographic assessment and confirmation of fracture was prompted by identification of unilateral swelling and/or limited range of motion of the affected limb, as well as those which raised clinical concern by healthcare providers prompting diagnostic imaging. Routine screening imaging for identification of non-symptomatic fractures was not conducted. LGEA patients who developed a symptomatic fracture were compared to those LGEA patients who did not develop a fracture.

### 2.2 Fracture protocol initiatives

A fracture-prevention protocol was initiated in 2012; incidence of fractures was evaluated pre and post-intervention. The fracture prevention protocol focused on the following initiatives: limiting medications detrimental to bone health; repleting Vitamin D both before and after paralysis; optimizing nutrients and minerals such as calcium and phosphorus in PN and in feeds; weekly labs during and after paralysis; multiple daily sessions with physical therapy (PT) for passive range of motion; educating family and care team about careful handling and fracture risk, and, instituting hospital-wide fracture precaution guidelines for during and after paralysis.

### 2.3 Statistical analysis

Variables were evaluated as potential risk factors for development of a fracture. Univariate analysis evaluated factors including gender, birth weight, preoperative gap length (cm), admission weight-for-age Z-score (WAZ), primary versus secondary Foker process (FP) patients, weight at Foker Stage I, days and episodes of paralysis, number of PN days, cumulative dose of loop diuretics adjusted for body weight and days exposed, and exposure to non-loop diuretics. Five covariates were further tested by multivariate logistic regression including: type of LGEA (Primary versus Secondary); number of unique episodes of paralysis; cumulative exposure to loop diuretics (furosemide equivalents/kg/day); exposure to non-loop diuretics; and exposure to fracture reduction protocol. Independent risk factors for the development of fractures were identified by the likelihood ratio test in multivariable logistic regression analysis with odds ratios and 95% confidence intervals (Hosmer et al., 2013). Statistical analysis was performed using IBM SPSS Statistics (version 21.0, IBM, Armonk, NY). Continuous data are mean ± standard deviation (SD) or median and interquartile range, and multivariate logistic regression was utilized to identify independent predictors of fracture (Vittinghoff and McCulloch, 2007). Two-tailed values of *P* < 0.05 were considered statistically significant.

## 3 Results

Fifty-nine patients met inclusion criteria and were included in the study. Twenty-three (39%) patients in the entire cohort incurred at least one fracture during the hospitalization utilizing the Foker process. This included 11 patients who suffered fractures to the humerus, 6 patients who suffered fractures to the femur, and 6 patients who suffered both. Given this high percentage in a specific population, a targeted fracture-prevention protocol was initiated in 2012. Fracture incidence decreased from 48% prior to the protocol to 21% following the protocol (*P* = 0.046) (see Table 1).

Baseline characteristics such as gender, birth weight, preoperative gap length, age at Foker process and exposure to PN were not risk factors in our cohort of patients for fractures. Univariate analysis identified several variables that increased risk for fractures, including secondary-FP patients (*P* = 0.008), number of paralytic episodes (*P* = 0.002), exposure to non-loop diuretics (*P* = 0.006), cumulative loop diuretic dose (*P* < 0.001), as well as cumulative loop diuretic over days exposed (*P* < 0.001). Outcome measures such as intensive care unit and total hospital stay (*P* < 0.001) were also significantly longer among patients with a fracture. Median ICU length of stay was 111 days (range: 73–217 days) in those with a fracture versus 62 days (range: 37–110 days) without a fracture (*P* = 0.002). Median total hospital length of stay was 155 days (range: 111–240 days) in those with a fracture versus 93 days (range: 63–147 days) (*P* < 0.001).

**Fig. 1:**
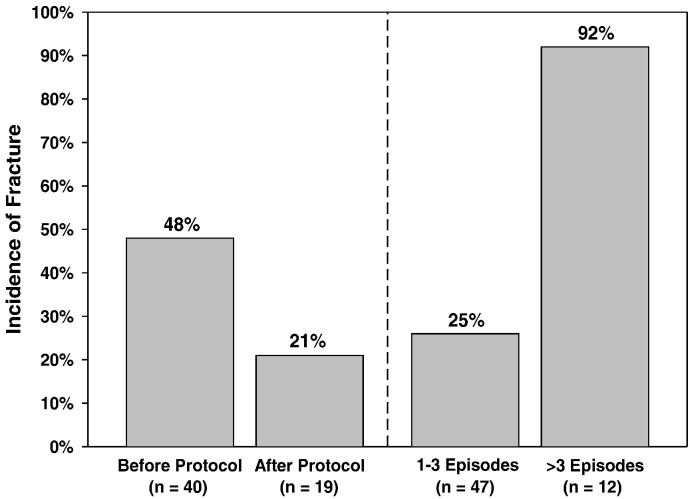
This figure illustrates the change in the incidence of symptomatic fractures among patients with Long-Gap Esophageal Atresia; it decreased significantly following implementation of a standardized protocol. This figure also denotes that the main risk factor for fractures was episodes of paralysis. Those who underwent 3 or more paralysis episodes during their Foker process had the highest risk for fracture.

Five covariates were tested by multivariable logistic regression analysis. This included the type of LGEA (Primary versus Secondary); number of unique episodes of paralysis; cumulative exposure to loop diuretics (furosemide equivalents/kg/day); exposure to non-loop diuretics; and exposure to fracture protocol. Table 2 confirmed that the number of times paralyzed was the only independent risk factor for the development of a fracture; patients with more than 3 unique episodes of paralysis incurred an estimated risk of fracture over 15 times higher than those patients paralyzed only once or twice (OR 15.87, 95% CI: 1.47–171.23, *P* = 0.008) (see Fig. 1). Multivariable analysis indicated that type of LGEA (*P* = 0.87), cumulative loop diuretic equivalent exposure (*P* = 0.29), exposure to non-loop diuretics (*P* = 0.38), and exposure to fracture protocol (*P* = 0.70) were not significant independent risk factors associated with the development of a fracture as seen in Table 2.

## 4 Discussion

Bone health has not previously been well-described in patients with LGEA. We identified a significant incidence of fractures in our cohort of infants with LGEA who underwent the Foker process. Multiple episodes of paralysis was the primary factor associated with highest risk of fractures in our cohort, albeit the small number of cases overall. The specific etiology of fractures in each individual LGEA patient remains unknown; however, the three major factors that likely contributed were: (a) prolonged immobilization due to pharmacological muscle relaxation, (b) suboptimal nutritional status due to need for parenteral nutrition, and (c) relatively high utilization of diuretics. Specific targeted interventions seemed to significantly decrease incidence of fractures in this high-risk population including optimizing nutritional status prior to initiating and during the Foker process, utilizing fracture precautions and incorporating PT while paralyzed, and limiting medications detrimental to bone health.

Given that our sample size was smaller following implementation of a risk-stratified fracture prevention program and we had less secondary LGEA patients, we will continue to monitor its effectiveness and adjust the protocol as our population increases. In particular, we must temper our overall results given (1) the difficulty of comparing patients with a seemingly unequal baseline fracture risk; and, (2) the recognition of the statistical limitation of interpreting the large confidence interval for the variable “number of times paralyzed”. Our data does suggest that bone health and metabolism should be an important clinical topic in the perioperative management of LGEA patients, which is similar to contemporary studies in other high-risk groups; the large number of absolute fractures suggests it to be clinically relevant topic (Khan et al., 2015, Theintz et al., 1992, Pironi et al., 2002, Done, 2012).

Bone growth and shape is determined by forces that are both intrinsic to the tissues themselves, as well as extrinsic physical forces (Rodriguez et al., 1988, Dunne and Clarren, 1986, Le Veau and Bernhardt, 1984, Smith, 1981). Mechanical signals modify bone mass and influence overall skeletal development, especially in children during periods of rather rapid growth, modeling and subsequent remodeling (Rodriguez et al., 1988, Dunne and Clarren, 1986, Le Veau and Bernhardt, 1984, Smith, 1981, Frost, 1987a, Frost, 1987b, Ehrlich and Lanyon, 2002). Our population is immobilized during the Foker process in order to aid with esophageal growth but this may have placed some of these children at higher risk for fractures given relative immobility and lack of extrinsic forces. In our study, number of paralysis episodes remained the only independent risk factor for fracture on multivariate analysis which suggests that immobilization may play a significant role in the pathogenesis of bone fragility in this population and may also be a surrogate marker for increased disease severity. Relative nutritional deficiencies and diuretic use also likely contribute to poor bone health. For example, children with intestinal failure may be at a particularly high risk for metabolic bone disease given factors as malabsorption, wasting of essential nutrients and minerals, and bacterial overgrowth (Khan et al., 2015, Klein et al., 1980, Ferrone and Geraci, 2007, Fewtrell, 2001). Such patients had a fracture risk of 29% but it was not correlated to previously identified risk factors (Khan et al., 2015). Similar to children with intestinal failure, our cohort did not have an increased risk of fractures with prolonged PN; however, unlike this cohort, WAZ scores were not predictive of an increased risk of poor bone homeostasis in our patient population, albeit none of the median WAZ scores were lower than − 2 (Khan et al., 2015, de Onis and Onyango, 2008).

Use and duration of diuretic have also been associated with an increased risk of fractures (Wei et al., 2012, Dokos et al., 2013, Kann et al., 2014). Exposure to both loop and non-loop diuretics was associated with increased risk of fracture on univariate analysis, though these covariates lost significance on the multivariable analysis. Patients with LGEA undergoing the Foker procedure often require significant exposure to both loop and non-loop diuretics; 27% of our patients sustained a fracture after being exposed concurrently to a non-loop diuretic. The concurrent use of both loop and non-loop diuretics may be confounded by a greater severity of illness and longer period of immobilization. Diuretics were not simply a surrogate marker for disease severity, as there have been multiple studies suggesting the pathological and pharmacological mechanism for increased fracture risk associated with the use of diuretics. In the case of loop diuretics, the pathological mechanism appears to be from the direct wasting of calcium from bones and the inhibition of laying down new bones, as well as it increases calcium in the urine and cause hypercalciuria, which can be detected by nephrocalcinosis (Schell-Feith et al., 2008). These revelations have made the LGEA team, as well as associated health care providers reassess patients who are exposed to both loop and non-loop diuretic therapy, as they still warrant close monitoring for fracture risk.

Limitations of this study were its retrospective nature based at a single institution with a relatively small cohort size. Still, our described cohort is one of the largest LGEA populations that underwent recent Foker anastomosis attempt, so our results should be generalizable to any center that utilizes the Foker process. The introduction of the risk-stratified fracture-reduction protocol was not the only variable that distinguished the pre and post implementation cohorts. There was also a trend towards fewer secondary repairs in the post-protocol cohort, a historically more complex cohort. We chose to test this variable along with the following other variables to identify independent predictors of fracture: number of unique episodes of paralysis; cumulative exposure to loop diuretics (furosemide equivalents/kg/day); exposure to non-loop diuretics; and exposure to fracture protocol. These variables were chosen given their clinical implications and the manner in which we could modify with our risk-stratified protocol. In spite of our concern that secondary LGEA patients might be a more “severe” cohort, this was not the case on multivariate analysis and the only variable that remained significant was number of episodes of paralysis.

## 5 Conclusions and future studies

Number of episodes of paralysis appears to be the main risk factor for fractures in this cohort of patients with LGEA. In the choice of diuretic therapy, exposure as to both loop and non-loop diuretics concurrently had an increased fracture risk and these patients still warrant close monitoring. At our institution, these data have initiated multi-disciplinary discussions focusing on fracture prevention, leading to the development of a consensus-driven approach. Short-term results show that the incidence of symptomatic fractures has decreased from 48% to 21% following the implementation of a standardized protocol in this series of LGEA patients. We will continue to monitor the safety and effectiveness of this approach.

## Figures and Tables

**Table 1:** Univariate analysis of LGEA patients & possible risk factors associated with fracture.

Variable	Fracture (n = 23)	No fracture (n = 36)	*P* value
Gender			0.60
Male	13 (43%)	17 (57%)	
Female	10 (35%)	19 (65%)	
Birth weight (kg)	2.5 ± 0.8	2.5 ± 0.8	0.83
Weight for age Z-score (WAZ)	− 1.6 ± 1.8	− 1.4 ± 1.6	0.65
Preoperative gap length (cm)	4.9 ± 1.9	4.4 ± 1.1	0.28
Type of LGEA			0.008⁎
Primary	7 (23%)	24 (77%)	
Secondary	16 (57%)	12 (43%)	
Age at Foker I (months)	5 (3–9)	3 (2–5)	0.17
Number of PN days	39 (21–77)	32 (22–47)	0.20
Number of times paralyzed	2 (1–5)	1 (1–1)	0.002⁎
Episodes of paralysis			< 0.001⁎
1–3	12 (25%)	35 (75%)	
> 3	11 (92%)	1 (8%)	
Loop diuretic exposure (days)	41 (17–129)	11 (5–15)	< 0.001⁎
Cum. loop diuretic equivalent (E/kg)	55.1 (21.5–404.8)	12.0 (4.1–17.6)	< 0.001⁎
Cum. loop diuretic equivalent (E/kg/days)	1.38 (1.20–2.62)	1.07 (0.96–1.31)	< 0.001⁎
Exposure to non-loop diuretic			0.006⁎
Yes	9 (27%)	24 (73%)	
No	14 (67%)	7 (33%)	
Fracture protocol exposure			0.046⁎
Yes	4 (21%)	15 (79%)	
No	19 (48%)	21 (52%)	

LGEA: Long-Gap Esophageal Atresia; PN: parenteral nutrition; ICU: intensive care unit; LOS: length of stay. Continuous data are mean ± standard deviation or median (interquartile range).

⁎ Statistically significant association.

**Table 2:** Independent risk factors of fracture based on multivariable logistic regression analysis.

Variable tested	Odds ratio	95% CI	*P* value
Type of LGEA (Secondary vs. Primary)	1.14	0.25–5.07	0.87
Number of times paralyzed (> 3 vs. 1–3)	15.87	1.47–171.23	0.008⁎
Cum. loop diuretic equivalent exposure (E/kg/days)	1.15	0.78–1.65	0.29
Exposure to non-loop diuretic	2.07	0.43–10.04	0.38
Fracture protocol exposure	0.72	0.14–3.68	0.70

LGEA: Long-Gap Esophageal Atresia; CI: confidence interval.

⁎ Statistically significant independent risk factor.
